# Deep learning-based amyloid PET positivity classification model in the Alzheimer’s disease continuum by using 2-[^18^F]FDG PET

**DOI:** 10.1186/s13550-021-00798-3

**Published:** 2021-06-10

**Authors:** Suhong Kim, Peter Lee, Kyeong Taek Oh, Min Soo Byun, Dahyun Yi, Jun Ho Lee, Yu Kyeong Kim, Byoung Seok Ye, Mi Jin Yun, Dong Young Lee, Yong Jeong, the Alzheimer’s Disease Neuroimaging Initiative, the KBASE Research Group

**Affiliations:** 1 Graduate School of Medical Science and Engineering, Korea Advanced Institute of Science and Technology (KAIST), Daejeon, Republic of Korea; 2 Department of Bio and Brain Engineering, Korea Advanced Institute of Science and Technology (KAIST), 291 Daehak-ro, Yuseong-gu, Daejeon, 34141 Republic of Korea; 3 Korea Advanced Institute of Science and Technology (KAIST), KI for Health Science Technology, Daejeon, Republic of Korea; 4 Department of Medical Engineering, Yonsei University College of Medicine, Seoul, Republic of Korea; 5 Department of Nuclear Medicine, Yonsei University College of Medicine, 50-1 Yonsei-ro, Seodaemun-gu, Seoul, 03722 Republic of Korea; 6 Department of Neurology, Yonsei University College of Medicine, Seoul, Republic of Korea; 7 Department of Neuropsychiatry, Seoul National University Bundang Hospital, Seongnam, Republic of Korea; 8 Institute of Human Behavioral Medicine, Medical Research Center, Seoul National University, Seoul, Republic of Korea; 9 Department of Neuropsychiatry, National Center for Mental Health, Seoul, Republic of Korea; 10 Department of Nuclear Medicine, SMG-SNU Boramae Medical Center, Seoul, Republic of Korea; 11 Department of Psychiatry, Seoul National University College of Medicine, 101 Daehak-ro, Joungno-gu, Seoul, 03080 Republic of Korea; 12 Department of Neuropsychiatry, Seoul National University Hospital, Seoul, Republic of Korea

**Keywords:** Alzheimer’s disease, Amyloid, Dementia, 2-[^18^F]FDG PET, Deep learning, Classification model

## Abstract

**Background:**

Considering the limited accessibility of amyloid position emission tomography (PET) in patients with dementia, we proposed a deep learning (DL)-based amyloid PET positivity classification model from PET images with 2-deoxy-2-[fluorine-18]fluoro-D-glucose (2-[^18^F]FDG).

**Methods:**

We used 2-[^18^F]FDG PET datasets from the Alzheimer's Disease Neuroimaging Initiative and Korean Brain Aging Study for the Early diagnosis and prediction of Alzheimer’s disease for model development. Moreover, we used an independent dataset from another hospital. A 2.5-D deep learning architecture was constructed using 291 submodules and three axes images as the input. We conducted the voxel-wise analysis to assess the regions with substantial differences in glucose metabolism between the amyloid PET-positive and PET-negative participants. This facilitated an understanding of the deep model classification. In addition, we compared these regions with the classification probability from the submodules.

**Results:**

There were 686 out of 1433 (47.9%) and 50 out of 100 (50%) amyloid PET-positive participants in the training and internal validation datasets and the external validation datasets, respectively. With 50 times iterations of model training and validation, the model achieved an AUC of 0.811 (95% confidence interval (CI) of 0.803–0.819) and 0.798 (95% CI, 0.789–0.807) on the internal and external validation datasets, respectively. The area under the curve (AUC) was 0.860 when tested with the model with the highest value (0.864) on the external validation dataset. Moreover, it had 75.0% accuracy, 76.0% sensitivity, 74.0% specificity, and 75.0% F1-score. We found an overlap between the regions within the default mode network, thus generating high classification values.

**Conclusion:**

The proposed model based on the 2-[^18^F]FDG PET imaging data and a DL framework might successfully classify amyloid PET positivity in clinical practice, without performing amyloid PET, which have limited accessibility.

**Supplementary Information:**

The online version contains supplementary material available at 10.1186/s13550-021-00798-3.

## Background

Alzheimer’s disease (AD) is characterized by the accumulation of β-amyloid (Aβ) and tau proteins. Aβ can be measured in humans with specific position emission tomography (PET) tracers or an examination of the cerebrospinal fluid (CSF) [1–3]. Patients clinically diagnosed with mild cognitive impairment (MCI) or AD have been found Aβ-negative [4, 5], thus leading to a pathophysiology-based unbiased and descriptive amyloid, tau, and neurodegeneration classification [6, 7]. The aforementioned criterion considers the Alzheimer’s continuum only when the Aβ marker is positive. However, high cost and low availability make it difficult to conduct amyloid PET. Furthermore, the limitations of CSF examination can be attributed to its invasiveness, thus necessitating alternative ways to classification amyloid status.

2-deoxy-2-[fluorine-18]fluoro-D-glucose (2-[^18^F]FDG) is the most widely used PET tracer for measuring brain metabolism, which is related to neuronal activity [8]. Studies using 2-[^18^F]FDG PET images reported on lesser hypometabolism in the bilateral temporoparietal regions and hippocampus in Aβ-negative participants with MCI and AD, compared to their positive counterparts [4]. There had been several attempts to develop an amyloid status classification model using magnetic resonance imaging (MRI), neuropsychological, or laboratory tests [9–11]. However, the performance was unsatisfactory. No attempts have been made using 2-[^18^F]FDG PET images.

Deep learning (DL) is the state-of-the-art mathematical algorithms that enable computers to automatically find patterns in large datasets. DL is studied in the medical imaging field for classification (diagnosis) [12, 13], predict prognosis [14, 15], detection [16, 17], and segmentation [18, 19]. A convolutional neural network (CNN), a subset of DL [20], is a DL model mimicking visual recognition concept which can extract features that reflect spatial relationships by applying non-linear convolutional filters. These spatial features pass through artificial neurons whose weights in the layers are properly set during training. Through this process, the model automatically learns hidden representative features from images and labels. 2-D-based deep learning architectures for 3-D medical images had been proposed: grid method (single montage image made by 16 images [21]), surface projection method (volumetric information projected onto a surface [22, 23]), and 2.5-D model (three axes (axial, coronal, and sagittal) images of brain volume [24]).

We aimed to construct a model that classifies amyloid PET positivity using 2-[^18^F]FDG PET that reflects brain metabolism and CNN architecture. In addition, we intended to validate the model using an independent external dataset with various diseases and classification probability analysis of submodules.

## Methods

### Study participants and data collection

**Fig. 1:**
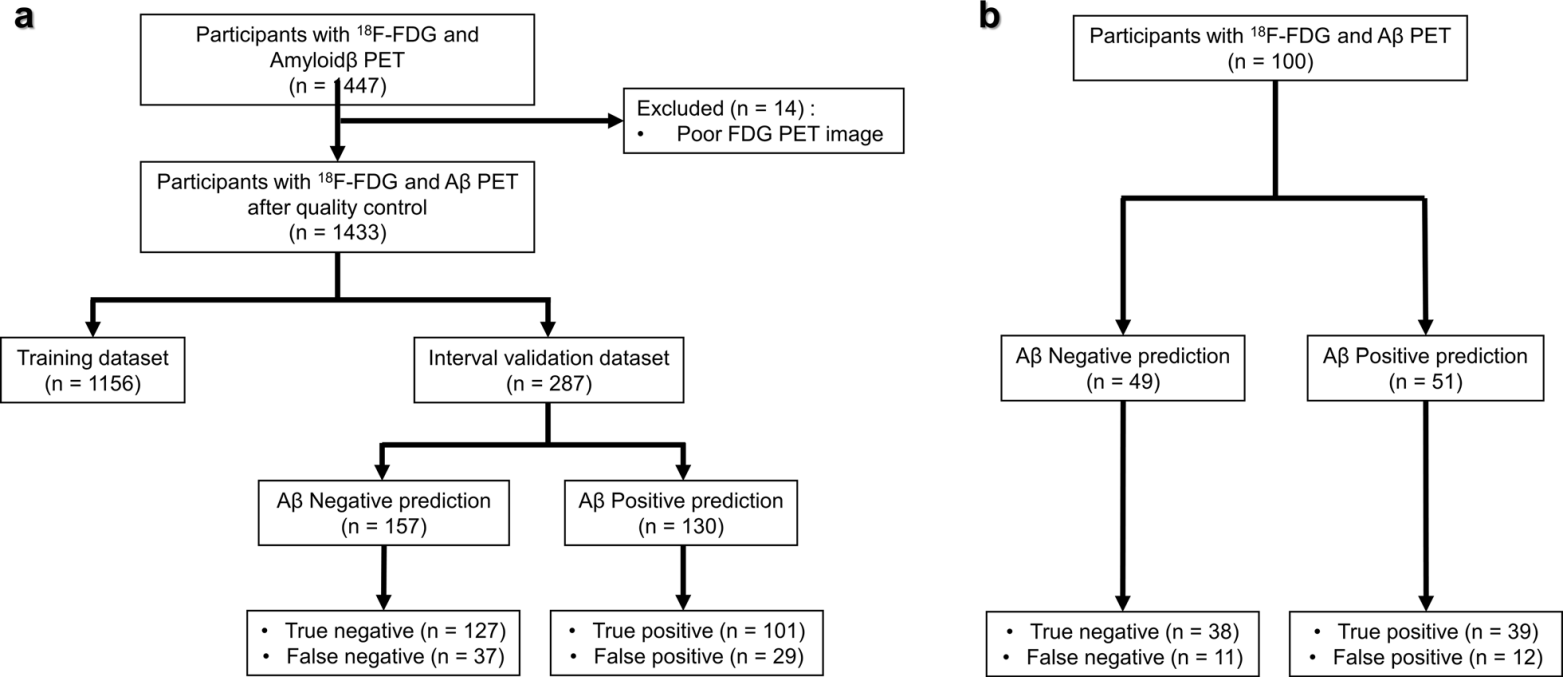
Flowchart of participants through the study for the **a** training and interval validation datasets, as well as **b** the external validation dataset. Abbreviation: FDG = fluorodeoxyglucose; Aβ = β-amyloid; PET = positron emission tomography

The following three datasets were used for model training, internal validation, and external validation: (i) Alzheimer’s Disease Neuroimaging Initiative (ADNI; adni.loni.usc.edu)-1, ADNI-GO (Grand Opportunities), ADNI-2, and ADNI-3 dataset [25], (ii) Korean Brain Aging Study for the Early diagnosis and prediction of Alzheimer’s disease (KBASE; kbase.kr) dataset [26], and (iii) dataset from the Severance Hospital. The inclusion criterion comprised individuals who underwent T1-weighted MRI, 2-[^18^F]FDG PET, and amyloid PET imaging. MRI and PET assessments were performed within 6 months. We excluded 11 and three participants from the ADNI and KBASE datasets, respectively, due to poor image quality. We eventually selected 963 and 470 participants from the ADNI and KBASE datasets, respectively (Fig. 1a). All data used in this study were from the baseline assessments. An additional 100 participants were recruited at the memory disorder clinic in the Department of Neurology at the Severance Hospital in Seoul, South Korea, between December 2017 and April 2019. The same criterion had been applied to ensure consistency (Fig. 1b). Table 1 summarizes the patient demographics and other information.

**Table 1:** Dataset demographics and clinical information

Category	CU (*n* = 271)		MCI (*n* = 550)		Dementia (*n* = 142)	
	Aβ –	Aβ +	Aβ –	Aβ +	Aβ –	Aβ +
**ADNI (*n* = 963)**						
Number of participants	179	92	238	312	17	125
Age, mean years (SD)	75.1 (7.0)	77.7 (5.7)	71.5 (8.4)	74.1 (7.2)	77.0 (8.2)	74.3 (8.2)
Gender: female, *n* (%)	83 (46.4)	54 (58.7)	104 (43.7)	134 (42.9)	2 (11.8)	57 (45.6)
Education: mean years (SD)	16.8 (2.6)	16.0 (2.7)	16.3 (2.5)	15.9 (2.9)	16.7 (2.4)	15.6 (2.7)
MMSE mean (SD)	29.1 (1.2)	28.5 (1.8)	28.2 (2.2)	26.9 (3.2)	23.2 (2.0)	23.0 (2.2)

Category	CU (*n* = 263)		MCI (*n* = 133)		Dementia (*n* = 74)	
	Aβ –	Aβ +	Aβ –	Aβ +	Aβ –	Aβ +
**KBASE (*n* = 470)**						
Number of participants	244	39	71	62	16	58
Age, mean years (SD)	68.3 (8.1)	74.2 (6.4)	73.5 (7.4)	72.9 (6.7)	75.4 (7.8)	72.4 (7.8)
Gender: female, *n* (%)	115 (51.3)	16 (41.1)	46 (64.8)	41 (66.1)	13 (81.2)	37 (54.4)
Education: mean years (SD)	11.7 (4.8)	12.2 (4.6)	9.5(4.6)	10.5 (4.5)	6.7 (5.2)	9.9 (5.2)
MMSE mean (SD)	26.9 (2.6)	27.2 (2.2)	23.2 (2.8)	21.5 (3.2)	16.5 (4.0)	16.9 (4.1)

Category	CU (*n* = 1)		MCI (*n* = 75)		Dementia (*n* = 24)	
	Aβ –	Aβ +	Aβ –	Aβ +	Aβ –	Aβ +
**University Hospital Dataset (*n* = 100)**						
Number of participants	1		38	37	11	13
Age, mean years (SD)	62 (0)		73.1 (6.1)	72.7 (6.8)	72.0 (4.6)	74.6 (5.7)
Gender: female, n (%)	1 (100)		19 (50.0)	21 (56.8)	6 (54.5)	10 (76.9)
Education: mean years (SD)	N/A	N/A	N/A	N/A	N/A	N/A
MMSE mean (SD)	28 (0.0)		24.7 (4.0)	23.8 (4.0)	21.7 (4.8)	18.2 (3.8)

Aβ = β-amyloid; CU = Cognitive unimpaired; MCI = Mild Cognitive Impairment; MMSE = Mini-Mental State Examination

While 80% of the ADNI and KBASE datasets was used for model training, the remaining 20% was used for internal validation. The Severance Hospital dataset was used for externally validating the classification model.

### Standard protocol approvals, registrations, and patient consent

The ADNI study protocol was approved by the institutional review board of each participating ADNI site (adni.loni.usc.edu/wpcontent/uploads/how_to_apply/ADNI_Acknowledgement_List.pdf). All participants provided written informed consent at the time of their enrollment in our study.

The use of the others datasets was approved by the Institutional Review Boards of the Seoul National University Hospital and the Severance Hospital. All participants provided their written informed consent.

### Imaging acquisition and preprocessing

The detailed 2-[^18^F]FDG /^18^F-florbetapir PET imaging protocol for the ADNI dataset is described at adni.loni.usc.edu/method/documents/. All participants underwent simultaneous PET and MRI scans using a 3T Biograph mMR (PET-MR) scanner (Siemens, Washington DC, USA) for the KBASE dataset. The procedure was performed based on the guidelines approved by the manufacturer. 3-D T1-weighted images with 3-D T1-weighted magnetization-prepared rapid acquisition with gradient echo (MPRAGE) sequence were acquired in the sagittal orientation with the following acquisition parameters: repetition time (TR) = 1670 ms, echo time (TE) = 1.89 ms, field of view (FOV) = 250 mm, and a 256 × 256 matrix with a 1.0 mm slice thickness. For the [^11^C] Pittsburgh compound-B (PiB) PET, we obtained a 30-min emission, 40-min after the intravenous administration of 555 MBq ^11^C-PiB. In contrast, for the 2-[^18^F]FDG PET scans, we requested the participant to fast for at least 6 h, prior to receiving the intravenous administration of 3.7 MBq/kg 2-[^18^F]FDG. Following the intravenous injections, the participants rested for 40 min in a dimly lit waiting room, prior to scanning. We processed the images for routine corrections and reconstructed them into a 256 × 256 image matrix using iterative methods (six iterations with 21 subsets) [26].

The dataset of the Severance Hospital included all participants who underwent MRI scans using a 3T Achieva scanner (Philips Medical System, Best, The Netherlands). We acquired a 3-D T1-weighted MRI sequence with a 3-D T1-turbo field echo sequence in the axial orientation with the following acquisition parameters: TR = 73,421 ms, TE = 5.09 ms, FOV = 215 mm, and a 1024 × 1024 matrix with a 1.0 mm slice thickness. We used a Discovery 600 scanner (GEHealthcare, Milwaukee, WI, USA) for the ^18^F-florbetaben PET scan. Moreover, we obtained a 20-min emission scan after the intravenous administration of 300 MBq ^18^F-florbetaben. The images were processed for routine corrections and reconstructed into a 256 × 256 image matrix using iterative methods (four iterations with 32 subsets).

### Data preprocessing

We conducted the preprocessing steps using SPM12 (Wellcome Trust Centre for Neuroimaging, University College London) and MATLAB R2019a (MathWork, Natick, MA). The amyloid and 2-[^18^F]FDG images were coregistered onto T1-weighted images and normalized into the Montreal Neurological Institute (MNI) template (McGill University, Montreal, Canada). Furthermore, we normalized each voxel of the 2-[^18^F]FDG PET image according to the mean intensity of the pons [27]. The pons were used as reference sites and extracted using an Automated Anatomical Labeling (AAL) template.

### Decision of Aβ PET status

We downloaded the UC Berkeley ^18^F-florbetapir analysis data from the ADNI dataset. Moreover, we classified each participant as Aβ-positive PET scan on observing a global standardized uptake value ratio (SUVR) > 1.11 [28].

We extracted the mean regional ^11^C-PiB uptake values from the frontal, posterior cingulate-precuneus, and lateral temporal and lateral parietal cortices using the individual AAL atlas from T1-coregistered ^11^C-PiB PET images for the KBASE dataset [29, 30]. In addition, we calculated the SUVRs for each region of interest (ROI) by dividing the mean value for all voxels within each ROI by the mean cerebellar uptake value. This can be attributed to its relatively low Aβ deposition [31]. We classified each participant as Aβ-positive PET scan if the SUVR was > 1.4 in at least one of the four ROIs [32].

We eventually analyzed the Severance Hospital dataset using a method similar to the aforementioned ones. We extracted the mean regional ^18^F-florbetaben uptake values from the frontal, anterior/posterior cingulate, lateral parietal, and lateral temporal cortices using the individual AAL atlas from T1-coregistered ^18^F-florbetaben PET images. Moreover, we calculated the SUVRs for each ROI by dividing the mean value for all voxels within each ROI by the mean cerebellar uptake value. We classified each participant as Aβ-positive PET scan if the SUVR was > 1.478 in at least one of the four ROIs [33].

### Deep learning architecture

**Fig. 2:**
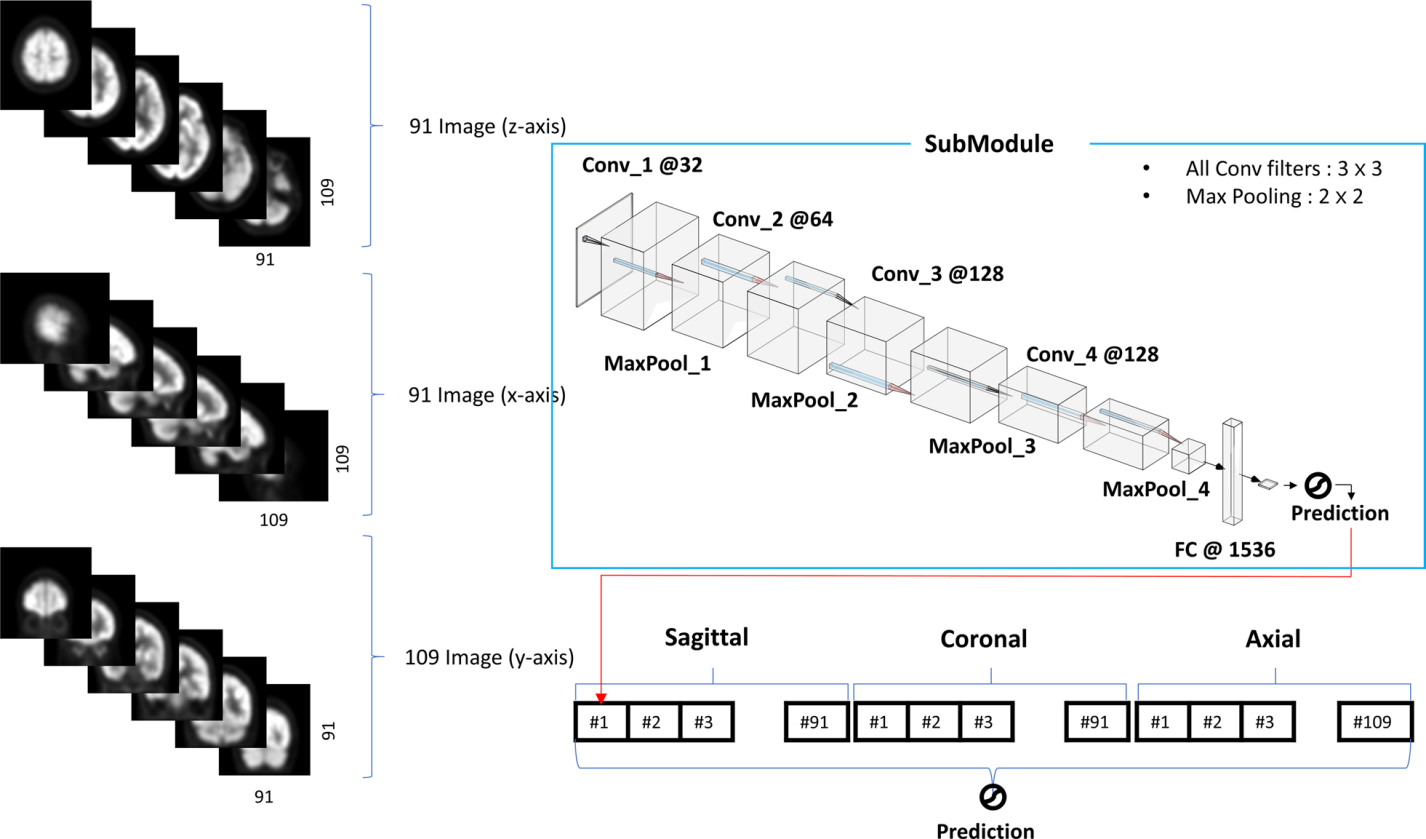
Convolutional neural network architecture in 2.5 dimensions (2.5-D). A total of 291 2-D images were used as inputs for the model. 291 number of prediction values obtained from 291 submodules pass through the fully connected layer for the final prediction. Abbreviation: Conv = convolution layer; MaxPool = Max *p*ooling layer; FC = fully connected layer

Initially, we tried to create a 3-D model for considering the axial, sagittal, and coronal spatial relationships. This can be attributed to the importance of spatial relationships in imaging. We have constructed two versions of 3-D CNN model (Additional file 1: Fig. 1). Nonetheless, the datasets were not large enough for model training. Furthermore, the 3-D models could not be trained even after augmenting the small datasets and needed a high computing power. Thus, we eventually built a 2.5- model that used the axial, coronal, and sagittal images as inputs to incorporate the 3-D spatial relationships. We entered a total of 291 images (91 images each along the z-axis and x-axis and 109 images along the y-axis) as inputs from each participant to the DL architecture (Fig. 2). The 2-D CNN submodule consists of four convolutional layers. The number of filters used in each convolutional layer was 32, 64, 128, and 128, respectively. Each convolutional layer had a kernel size of 3 × 3 and a stride of 1. Following the convolutional layers, we added a batch normalization layer and a rectified linear unit (ReLU). While we used the ReLU for the nonlinear activation function, the batch normalization layer improved the training convergence speed. We then added a 2 × 2 max pooling layer with a stride of 2 for down-sampling of the feature maps. After the four convolutional layers, we used a completely connected layer to determine the Aβ PET status in each image with sigmoid activation. DL architecture consists of 291 submodules, and each submodule had been assigned a different weight value. We concatenated 291 prediction values as inputs to the aforementioned layer. In addition, we used sigmoid activation as a function for the final prediction. When the final prediction value was larger than 0.5, the model classified this participant Aβ-positive PET scan. We initialized the 2-D convolutional filters and the completely connected layers using the He-weight initialization [34] and the Xavier initialization, respectively. Our architecture was trained using a mini-batch size of four and an Adam optimizer with a 0.0001 learning rate for a maximum of 30 epochs. We monitored the internal validation loss after every epoch. Moreover, we saved the model weights upon encountering the lowest internal validation loss. We used the DL model with the lowest internal validation loss to classify the amyloid PET positivity on the external validation dataset. All model was made in open-source package PyTorch on NVIDIA GTX 2080Ti.

### Performance evaluation

First, our model was trained on the ADNI and KBASE datasets. These datasets were randomly divided into two groups for training and internal validation. Moreover, our model was externally validated on the Severance Hospital dataset. This process was repeated 50 times.

We computed the receiver operating characteristic (ROC) curve, the area under curve (AUC) values, accuracy, sensitivity, and specificity of model classifications on all datasets to evaluate the performance of our model. In addition, we computed the F1-score, the harmonized mean of the positive predictive value and sensitivity.

The F1-score is defined as follows:

$${\text{F}}1{\text{ - score}} = \frac{2 \times True\;Positive\;Value}{{2 \times True\;Positive\;Value + False\;Positive\;Value + False\;Negative\;Value}}$$

In addition, performance of our model was evaluated within each subgroup, such as CU, MCI, and demented participants.

Second, to evaluate the model performance between two distinct datasets with different diagnoses, composition, and ethnicity, our model was trained and internally validated on the ADNI dataset, and externally validated on the KBASE dataset.

### Voxel-wise analysis

We conducted a two-sample t test to find substantial differences in glucose metabolism between the Aβ-positive and Aβ-negative PET scan participants. In addition, we conducted the vowel-wise analysis using SPM12 [35]. The threshold for statistical mapping was at FWE-corrected *p* < 0.05 or uncorrected *p* < 0.001. We applied the spatial clustering of regions with statistically relevant voxels using a clustering threshold of *k* > 50 voxels for eliminated voxel clusters with smaller sizes.

### Classification basis for understanding model decision

**Fig. 3:**
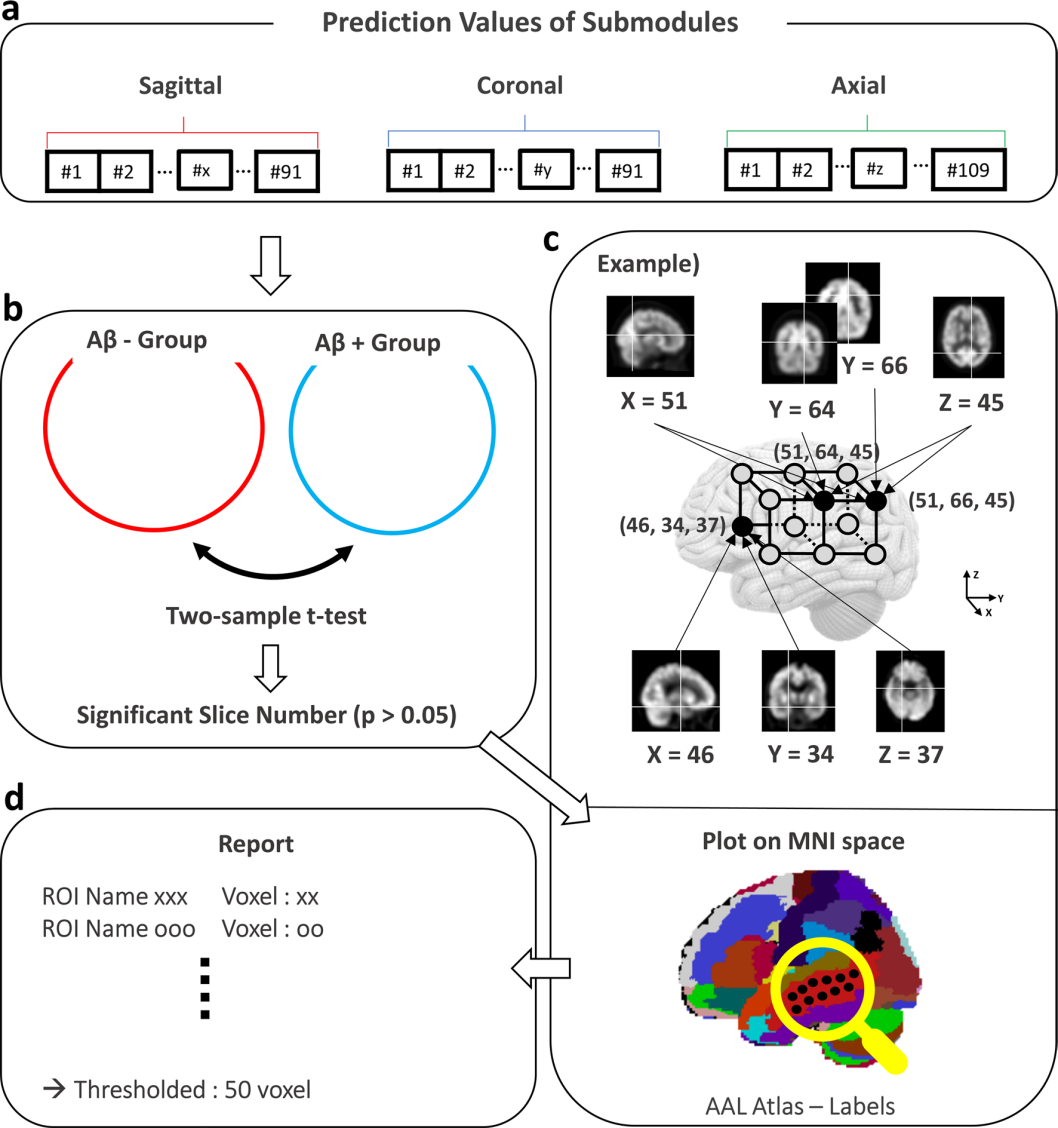
Analysis of prediction values from submodules to understand the model’s decision. **a** Acquiring prediction values from submodules along the 3 axes. **b** The Aβ-positive and Aβ-negative PET scan participants are divided based on the final classification, and a two-sample t test is performed to determine substantial slice numbers. c) Points are plotted for each slice number in the MNI space and correlated labels are extracted from the AAL atlas. In the example, if a significant slice number is *x* = 46, 51, *y* = 34, 64, 66, and *z* = 37, 45, a total of 12 points are plotted in the MNI space. However, actual significant points are (46,34,37), (51,54,45), and (51,66,45). d) To exclude unrelated points, ROIs in the AAL atlas with more than 50 points are used for comparison with the result of the voxel-wise analysis. Abbreviation: Aβ = β-amyloid; MNI = Montreal Neurological Institute; AAL = Automated Anatomical Labeling; ROI = region of interest

We commonly used a class activation map (CAM) to confirm the classification basis. Nonetheless, we could not apply it as our model was built using a 2.5-D CNN [36]. Thus, we suggested a method to approximately understand the decision basis of our model. Figure 3 illustrates the way of analysis of classification probability from the submodules. This facilitates the verification of the ROIs that contribute to our classification. First, we extracted the prediction values from the submodules. Second, we performed a two-sample t test based on the final classification between the Aβ-positive and Aβ-negative PET scan participants (*p* value below 0.05, two-tailed) and obtained a substantial slice number. Third, we considered the (*x*, *y*, *z*) point as statistically significant when the ‘*x*’th, ‘*y*’th, and ‘*z*’th planes of the sagittal, the coronal, and axial axis showed substantial differences. We eventually plotted the aforementioned points in the MNI space. If ×0, ×1 in the sagittal; y0, y1 in the coronal; and *z*0, *z*1 in the axial planes were significant slices, a total of 8 points could be plotted in the MNI space. Assuming that (× 0, *y*0, *z*0) and (× 1, *y*1, *z*1) are significant points among the plotted points, the unrelated six points ((× 0,*y*0,*z*1), (× 0,*y*1,*z*0), (× 0,*y*1,*z*1), (× 1,*y*0,*z*0), (× 1,*y*0,*z*1), (× 1,*y*1,*z*0)) may also be included in the MNI space. Therefore, to exclude these unrelated points, we finally extracted clusters with more than 50 points (same threshold as voxel-wise analysis) from the AAL atlas. Moreover, we compared the aforementioned regions with the results of our classification-based voxel-wise analysis.

## Results

### Participant demographics

Table 1 shows the demographic data of the participants in the three datasets. There were 686 out of 1433 (47.9%) and 50 out of 100 (50%) Aβ-positive PET scan participants in the ADNI/KABSE and Severance Hospital datasets, respectively.

### Performance of deep learning model

**Fig. 4:**
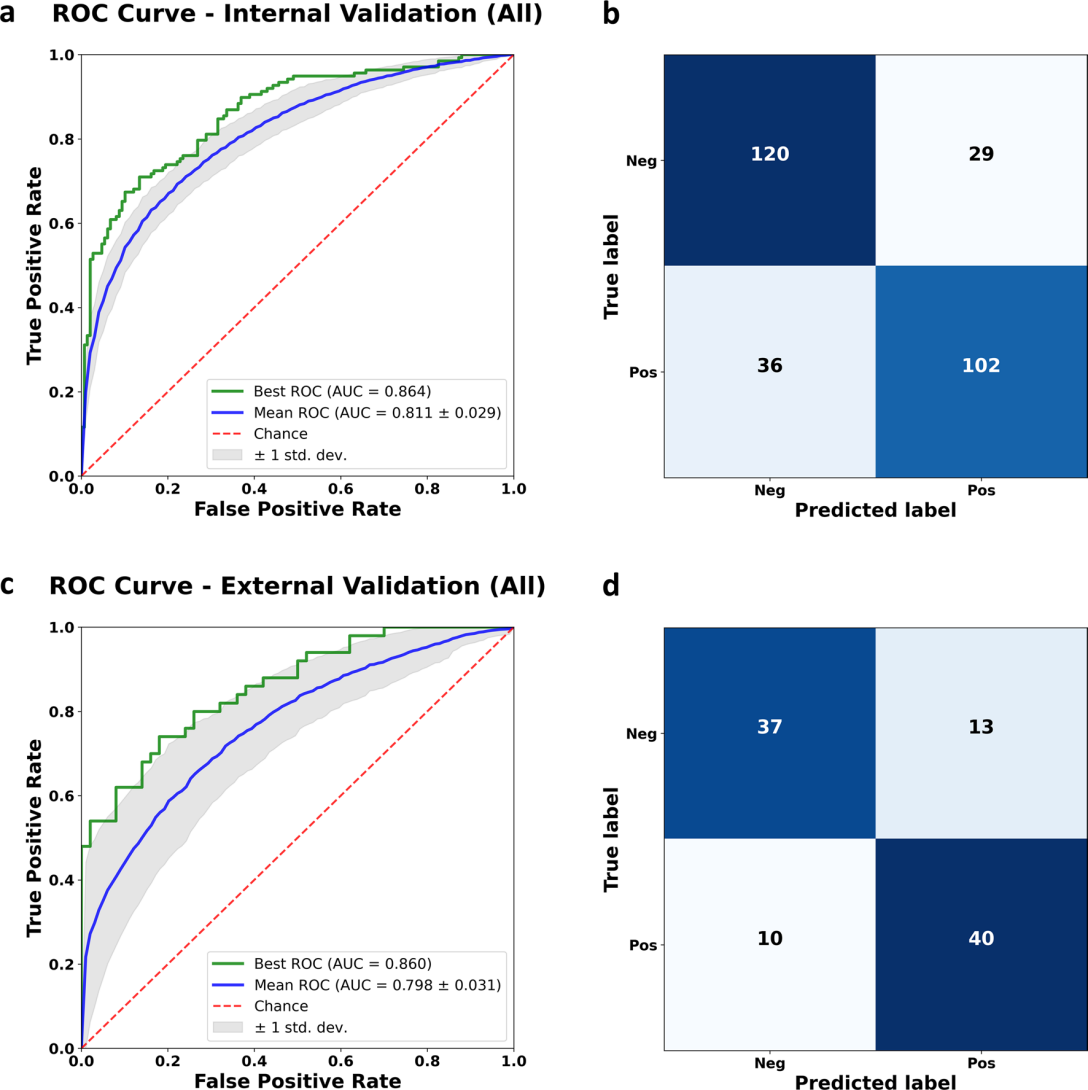
ROC curve and confusion matrix of the best performing model for classifying Aβ PET positivity in the internal validation dataset (**a, b**) and the external validation dataset (**c, d**). Abbreviation: ROC = receiver operating characteristic; AUC = area under curve

The internal validation dataset included 129 and 138 Aβ-negative and Aβ-positive PET scan participants (20% of ADNI/KBASE datasets), respectively. In contrast, the external validation dataset included 50 participants each in the abovementioned categories. The model achieved an AUC of 0.811 (95% confidence interval (CI) of 0.803–0.819) and 0.798 (95% CI, 0.789–0.807) on the internal and external validation datasets, respectively. Table 2 summarizes the accuracy, sensitivity, specificity, and F1 score. When we performed external validation with the model shown best AUC value (0.864) on the internal validation dataset, the AUC, accuracy, sensitivity, specificity, and F1 score of 0.860, 0.770, 0.800, 0.740, and 0.780, respectively. Figure 4 shows the ROC curves and confusion matrix of the aforementioned model for classifying Aβ PET positivity in the internal and external validation datasets.

**Table 2:** Classification performance for Aβ PET positivity on ADNI, KBASE, and Severance hospital datasets

	Mean (95% CI)		AUC value		Accuracy		Sensitivity		Specificity		F1-score
**Internal validation (ADNI and KBASE)**	All		0.811 (0.803, 0.819)		0.733 (0.726, 0.740)		0.678 (0.664, 0.691)		0.785 (0.771, 0.799)		0.709 (0.701, 0.717)
	CU		0.717 (0.705, 0.729)		0.748 (0.737, 0.758)		0.381 (0.352, 0.410)		0.870 (0.857, 0.883)		0.422 (0.398, 0.446)
	MCI		0.757 (0.746, 0.768)		0.682 (0.672, 0.692)		0.651 (0.636, 0.666)		0.718 (0.698, 0.738)		0.690 (0.680, 0.701)
	AD		0.816 (0.789, 0.843)		0.862 (0.848, 0.875)		0.950 (0.939, 0.960)		0.336 (0.283, 0.390)		0.921 (0.912, 0.929)
**External validation (Severance hospital)**	All		0.798 (0.789, 0.807)		0.690 (0.681, 0.699)		0.768 (0.748, 0.789)		0.612 (0.586, 0.639)		0.712 (0.703, 0.721)
	MCI		0.769 (0.762, 0.776)		0.698 (0.691, 0.705)		0.702 (0.688, 0.715)		0.694 (0.675, 0.714)		0.718 (0.711, 0.725)
	Demented participants		0.806 (0.803, 0.809)		0.599 (0.573, 0.624)		0.868 (0.850, 0.886)		0.462 (0.414, 0.509)		0.598 (0.587, 0.609)

Aβ = β-amyloid; CI = Confidence interval; AUC = Area under curve

Internal validation model performance by the subgroups (CU, MCI, and demented (AD)) were shown in the Additional file 1: Fig. 2. The model achieved an AUC of 0.717 (95% CI, 0.705–0.729), 0.757 (95% CI, 0.746–0.768) and 0.816 (95% CI, 0.789–0.843) on CU (*n* = 105), MCI (*n* = 141) and AD (*n* = 41) participants, respectively. When AUC value was 0.864 on internal validation dataset, the AUC were 0.738, 0.839 and 0.887 on CU, MCI, and AD, respectively. Moreover, external validation model performance by the subgroups were shown in the Additional file 1: Fig. 3. The model achieved an AUC of 0.798 (95% CI, 0.789–0.807) and 0.787 (95% CI, 0.774–0.800) on MCI (n = 75) and demented (*n* = 24) participants. When we performed external validation with the model that showed best AUC value (0.864) on the internal validation dataset, the AUC were 0.865 and 0.832 on MCI and demented participants, respectively. Table 2 summarizes the accuracy, sensitivity, specificity, and F1 scores.

Additional file 1: Table 1 shows model performance by all datasets and subgroups in the internal validation (part of the ADNI dataset) and external validation (KBASE dataset). The model achieved an AUC of 0.769 (95% CI, 0.762–0.776) and 0.806 (95% CI, 0.803–0.809) on the internal and external validation datasets, respectively. When we performed external validation with the model, it indicated a good AUC value (0.800) on the internal validation, the AUC previously was 0.822. The accuracy, sensitivity, specificity, and F1 score and values on subgroups are summarized in the Additional file 1: Table 1.

### Voxel-wise analysis

**Fig. 5:**
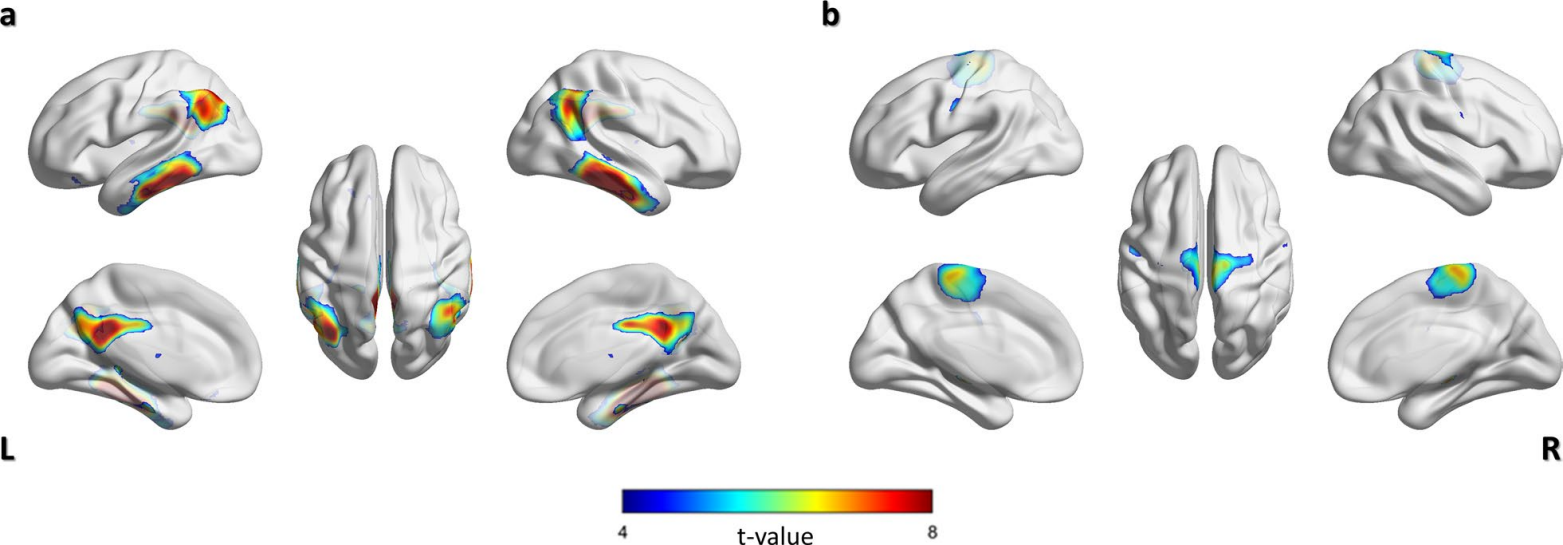
Result of voxel-wise 2-[^18^F]FDG PET analysis performed in the ADNI and KBASE datasets. a) Areas of higher glucose metabolism in Aβ-negative PET scan participants compared to Aβ-positive PET scan participants. b) Areas of higher glucose metabolism in Aβ-positive PET scan participants compared to Aβ-positive PET scan participants. All results are presented with a threshold of *p* < 0.05, FWE-corrected, and 50 voxels. Abbreviation: ADNI = Alzheimer’s Disease Neuroimaging Initiative; KBASE = Korean Brain Aging Study for the Early diagnosis and prediction of Alzheimer’s disease; FDG = fluorodeoxyglucose; PET = positron emission tomography; Aβ = β-amyloid

In the ADNI and KBASE datasets, Aβ-negative PET scan participants showed higher glucose metabolism than Aβ-positive PET scan participants at bilateral angular, supramarginal, precuneus, middle and posterior cingulate, inferior and middle temporal, parahippocampal, and fusiform gyri (Fig. 5a). In contrast, Aβ-positive PET scan participants showed higher glucose metabolism in the bilateral paracentral lobules, pre- and postcentral gyri (Fig. 5b).

### Classification basis for understanding model decision

**Fig. 6:**
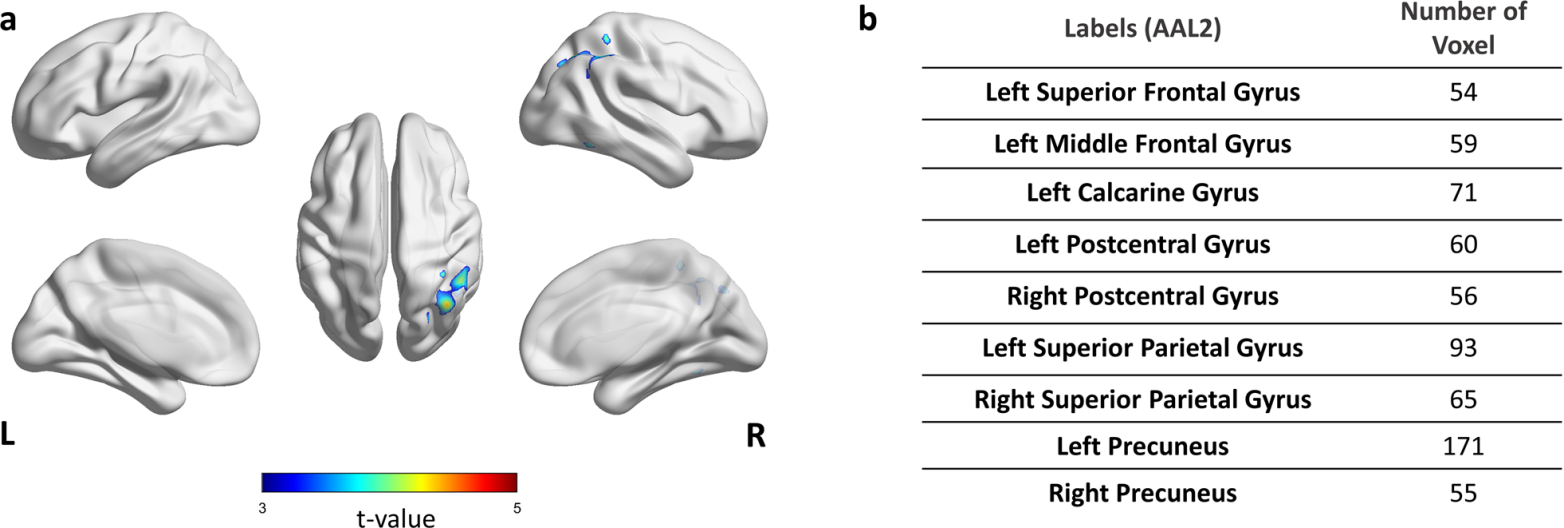
Result of voxel-wise analysis and analyzing prediction values from submodules in the external validation dataset. **a** Areas of higher glucose metabolism in Aβ-negative PET scan participants compared to Aβ-positive PET scan participants. The results are presented with a threshold of p < 0.001, uncorrected, and 50 voxels. **b** Analysis result of prediction values from submodules. Abbreviation: AAL = Automated Anatomical Labeling; Aβ = β-amyloid

Analyzing of the prediction values from the submodules derived the following nine ROIs from the external validation dataset: bilateral postcentral gyri, superior parietal gyri and precuneus, left superior frontal gyrus, middle frontal gyrus, and calcarine gyrus (Fig. 6b). The voxel-wise analysis of 2-[^18^F]FDG PET showed differences in the left inferior temporal, left middle temporal gyrus, right angular, right inferior parietal, right superior parietal, right middle occipital gyri, and right cuneus (Fig. 6a).

## Discussion

A sound understanding of amyloid pathology is critical for the accurate diagnosis and prognosis prediction in the Alzheimer’s disease continuum or in patients with dementia. The following two methods are currently available to examine amyloid pathology: amyloid PET and CSF analysis through lumbar puncture. However, amyloid PET is expensive and not universally available. Moreover, lumbar puncture is relatively invasive and poses potential side effects. Thus, the classification of amyloid pathology using long-established and widely distributed imaging techniques, such as MRI or 2-[^18^F]FDG PET, appears promising. Several studies have attempted to classify amyloid pathology by analyzing the cortical thickness from structural MRI (sMRI) [9, 10]. Nonetheless, sMRI has its limitations in early classification. This can be attributed to the occurrence of cortical thinning primarily at the late stage of the disease. Moreover, it does not provide any functional information. Glucose metabolism changes before a change in the cortical thickness in AD [37]. Hence, we assumed that 2-[^18^F]FDG PET would better classify amyloid pathology. However, the use of 2-[^18^F]FDG PET images for amyloid classification is rare. Thus, we developed a DL model that determines amyloid pathology from 2-[^18^F]FDG PET imaging data.

We found a lower glucose metabolism in the Aβ-PET-positive participants in the bilateral angular, supramarginal, precuneus, middle and posterior cingulate, inferior and middle temporal, parahippocampal, and fusiform gyri, consistent with previous reports (Fig. 4a) [4]. However, we recorded a higher glucose metabolism in the bilateral paracentral lobules and pre- and postcentral gyri in the Aβ-positive participants. According to Braak staging, the aforementioned regions are reportedly preserved during the progress of Aβ pathology [38]. These findings support the possibility of its classification power and form the basis of model interpretation.

Despite these possibilities, the datasets were extremely small for building a 3-D DL model. However, the model could extract more spatial relationships while considering the coronal and sagittal images, in addition to the axial images. Therefore, we designed a 2.5-D DL model for completely considering the 3-D spatial relationships. The CNN DL model can be usually explained using CAM for visualization. However, the last layer of each submodule gets replaced with a global average pooling layer during CAM. Moreover, the values from the submodules passed the fully connected layer for the final classification. This in turn leads to the disappearance of the location. Thus, the analysis would provide limited spatial information in 2.5-D. Therefore, we developed a 2.5-D method that analyzed the prediction values from the submodules to understand the basis of this decision.

We trained and internally validated the combined internal datasets. The internal validation results were impressive, with AUC values, accuracy, sensitivity, specificity, and F1 score of 0.811, 0.733, 0.678, 0.785, and 0.709, respectively. We encounter several diseases characterized by memory impairment in clinical practice. In contrast, the ANDI and KBASE datasets only contained the cognitive unimpaired and Alzheimer’s disease continuum. Thus, we externally validated our proposed model using a dataset, including various neurological diseases that cause memory impairment. The external validation dataset included clinically diagnosed AD, Lewy body dementia, epileptic cognitive disorder, normal pressure hydrocephalus, frontotemporal dementia, and vascular dementia. In addition, it included various clinical stages, such as one, 75, and 24 patients with cognitive unimpaired, MCI, and dementia, similar to the clinical situation. Moreover, the application of the proposed model to the external validation dataset also resulted in a high AUC value, accuracy, sensitivity, specificity, and F1 score of 0.798, 0.690, 0.768, 0.612, and 0.712, respectively. Furthermore, when we performed external validation with the model shown the best AUC value (0.864) on the internal validation dataset, the AUC value, accuracy, sensitivity, specificity, and F1 score was 0.860, 0.770, 0.800, 0.740, and 0.780, respectively. The performance of our model is comparable to that of the model based on longitudinal sMRI (AUC value was 0.86) [9]. However, it is better than that of the model which used patient demographics and sMRI (AUC value was 0.79) [10]. This enhanced performance could be attributed to the changes in glucose metabolism before cortical atrophy. Thus, our model that only used one-time 2-[^18^F]FDG PET images could be effectively used for classifying amyloid PET positivity in memory disorder clinics.

The performances by subgroups in the internal validation resulted in a high AUC of 0.717, 0.757, and 0.816 in the CU, MCI, and AD participants, respectively. Our model outperformed conventional models by producing comparatively better AUC in AD subgroup that indicated many significant differences within the group on voxel-wise analysis. Additionally, it resulted in a good AUC in CU subgroup that indicated a few significant differences within the group on voxel-wise analysis. Moreover, the application of the proposed model to the external validation dataset also resulted in a high AUC of 0.798 and 0.787 in the MCI and demented participants, respectively. These results were achieved because the external validation dataset contained many MCI and demented participants data. Additionally, the result of the internal validation in the Alzheimer’s Continuum was impressive, but the results for when this model was applied on the external validation dataset with various clinical diagnosis were impressive as well. This reflects the applicability in clinical practice.

When our model was trained and internally validated in the ADNI dataset and externally validated in the KBASE dataset, the model achieved the AUC values, accuracy, sensitivity, specificity, and F1 score were 0.769, 0.698, 0.702, 0.694, and 0.719, respectively. These results suggested that the performance was comparatively decent than when we used the ADNI and KBASE dataset for training and internal validation (Additional file 1: Fig. 4 and Additional file 1: Table 1). The reasons of the differences were suggested as follows: First, the ADNI and KBASE had different patients’ composition. Within the ADNI dataset, MCI participants were the most common, and 55% of the participants had amyloid PET-positive scan, while, within the KBASE, CU participants were the most common, and 34% of the participants had amyloid PET-positive scan. Second, the two datasets were obtained under different ethnicities, different amyloid tracers, and various PET manufacturers. Although we did spatial normalization with ethnicity specific template in the preprocessing pipeline, there may be remained differences. However, from the other perspective, the above results were also affirmative. The model with the best AUC value (0.800) in the internal validation showed good AUC values of 0.745 and 0.984 in MCI and AD, respectively, which also showed good AUC values of 0.837 and 0.721 in MCI and AD in the external validation, respectively. These results were encouraging since one could classify amyloid PET positivity status from the dataset obtained regardless of the ethnicities, tracers, and MRI manufacturers.

On internal and external validation, 10–15% of false negative and false positive cases were found. According to Alzheimer’s disease biomarker curve [39], amyloid deposition preceded the hypometabolism, which could explain the false negative cases. There are two speculation for comprehend false positive cases. First, approximately 10% of false negative cases are due to sensitivity of amyloid PET [40, 41]. Second, other cause of dementia, including Lewy body dementia, could be shown similar hypometabolism pattern [42].

The common region between the analysis of prediction value from the submodules and voxel-wise analysis included the right superior parietal gyrus in the external validation dataset (Fig. 6). However, we observed additional regions with different metabolism in the training and internal validation datasets that only included AD (Fig. 5). The above-mentioned difference could be attributed to the characteristics of the patient population, including only AD or other diseases. Therefore, our model might have extracted these hidden features and used them for the classification.

Our study had some limitations. First, we used several datasets that used different protocols and amyloid tracers for data collection. Moreover, these datasets included patients of various ethnicities. However, their characteristics suggest the general applicability of our model regardless of the aforementioned variabilities. Second, amyloid pathology could be detected by CSF Aβ_42_ or amyloid PET studies; most cases show concordant results in the Alzheimer’s disease continuum, whereas 6–21% show discordant results [43]. Both results should be considered for more accurate amyloid pathology; however, CSF Aβ_42_ was not available in all datasets. Especially in the ADNI dataset, only 66 participants underwent sMRI, 2-[^18^F]FDG PET, amyloid PET, and CSF Aβ_42_. We chose amyloid PET only because deep learning requires a large amount of data. Third, the DL framework had a black box limitation. Thus, the model visualization method, such as CAM, can further explain our classification model. We could not apply CAM because of the limitation of available datasets. In contrast, we used three axes images (2.5-D) as the inputs to improve the classification accuracy. The development of a 3-D DL model from larger datasets would facilitate the interpretation through CAM. Fourth, we suggested a novel method for the brief understanding of our model’s decision. After the *t* test between the amyloid PET-positive and PET-negative participants on SPSS, we obtained 20 slices in the sagittal plane, 27 slices in the coronal plane, and 24 slices in the coronal plane. We considered the (*x*, *y*, *z*) point as statistically significant when the ‘*x*’th, ‘y’th, and ‘z’th planes of the sagittal, coronal, and axial axes, respectively, showed substantial differences; a total of 12,960 (20×27×24) points were plotted in the MNI space. However, our method suffers from a limitation due to the following assumption. If the (*x*,*y*,*z*) point is significant, the ‘*x*’ th plane in the sagittal, ‘y’th plane in the coronal, and ‘*z*’th plane in the axial could be significant; however, the opposite was not assumed. Therefore, many unrelated points were plotted in the MNI space; ROIs clustered with more than 50 points were extracted to remove these unrelated points. Further investigation is required to accurately understand the model’s decision. Fifth, we did not include other biomarkers or neuropsychological test results. An incorporation of these features might have improved the performance. However, we tried to build a classification model by only using images, which can be applied more generally.

In conclusion, we proposed a DL model that can classify the amyloid PET positivity from 2-[^18^F]FDG PET imaging and demonstrated its high performance across an external test dataset. A large-scale external validation of multi-institutional data, model calibration, and optimization of sensitivity needs to be incorporated into the clinical workflow. The aforementioned model can serve as an important decision supporting tool to aid clinicians while classifying amyloid PET positivity.

## Conclusion

The proposed model based on the 2-[^18^F]FDG PET imaging data and a DL framework might successfully classify amyloid PET positivity in clinical practice, without performing amyloid PET, which have limited accessibility.

## Supplementary Information

**Additional file 1**. **Supplementary Fig. 1.** Convolutional neural network architecture in 3 dimensions. (3-D) **a**) Custom ResNet with 15 layers and 4 residual layers. **b**) A structure that simply changed our 2.5-D architecture to 3-D. This consist of 4 convolution layers. Abbreviation: Conv = convolution layer; BN = batch normalization; ReLU = rectified linear unit; @ = number of channels; MaxPool = Max pooling layer; AvgPool = average pooling; FCN = fully connected layer. **Supplementary Fig. 2.** ROC curve and confusion matrix by the subgroups (CU, MCI, and AD participants) in the interval validation. (**a**, **b**) CU participants, (**c**, **d**) MCI participants and (**e**, **f**) AD participants. Abbreviation: ROC = receiver operating characteristic; AUC = area under curve, CU = cognitively unimpaired, MCI = mild cognitive impairment, AD = Alzheimer’s dementia. **Supplementary Fig. 3.** ROC curve and confusion matrix by the subgroups (MCI and demented participants) in the external validation. (**a**, **b**) MCI participants and (**c**, **d**) demented participants. Abbreviation: ROC = receiver operating characteristic; AUC = area under curve, MCI = mild cognitive impairment. **Supplementary Fig. 4.** (**a**-**d**) ROC curve and confusion matrix by all datasets and subgroups in the internal validation (part of the ADNI dataset). **a**) all participants, **b**) CU participants, **c**) MCI participants, and **d**) AD participants. (**e**-**h**) ROC curve and confusion matrix by all datasets and subgroups in the external validation (the KBASE dataset). **e**) all participants, **f**) CU participants, **g**) MCI participants, and **h**) AD participants. Abbreviation: ROC = receiver operating characteristic; AUC = area under curve, CU = cognitively unimpaired, MCI = mild cognitive impairment, AD = Alzheimer’s dementia. **Supplementary table 1.** Classification performance for Aβ PET positivity on ADNI and KBASE datasets.

## Data Availability

All imaging, and demographics data used in this article from ADNI are available and were downloaded from the ADNI website (adni.loni.usc.edu). Upon request, we will provide a list of ADNI participants for replication purposes. The data for this analysis are owned by the KBASE research group. Requests for data access can be submitted to the administrative coordinator of the group by e-mail (kbasecohort@gmail.com).

## Code availability

Request for code access can be submitted to the author by e-mail (drshkim@kaist.ac.kr).
