# Supplementary material for: Deep learning-based amyloid PET positivity classification model in the Alzheimer’s disease continuum by using 2-[18F]FDG PET
Source: EJNMMI Res. 2021 Jun 10;11:56. doi: 10.1186/s13550-021-00798-3 (PMC8192639; doi:10.1186/s13550-021-00798-3)
Supplement: Supplementary file 1 — Additional file 1. Supplementary Fig. 1. Convolutional neural network architecture in 3 dimensions. (3-D) a) Custom ResNet with 15 layers and 4 residual layers. b) A structure that simply changed our 2.5-D architecture to 3-D. This consist of 4 convolution layers. Abbreviation: Conv = convolution layer; BN = batch normalization; ReLU = rectified linear unit; @ = number of channels; MaxPool = Max pooling layer; AvgPool = average pooling; FCN = fully connected layer. Supplementary Fig. 2. ROC curve and confusion matrix by the subgroups (CU, MCI, and AD participants) in the interval validation. (a, b) CU participants, (c, d) MCI participants and (e, f) AD participants. Abbreviation: ROC = receiver operating characteristic; AUC = area under curve, CU = cognitively unimpaired, MCI = mild cognitive impairment, AD = Alzheimer’s dementia. Supplementary Fig. 3. ROC curve and confusion matrix by the subgroups (MCI and demented participants) in the external validation. (a, b) MCI participants and (c, d) demented participants. Abbreviation: ROC = receiver operating characteristic; AUC = area under curve, MCI = mild cognitive impairment. Supplementary Fig. 4. (a-d) ROC curve and confusion matrix by all datasets and subgroups in the internal validation (part of the ADNI dataset). a) all participants, b) CU participants, c) MCI participants, and d) AD participants. (e-h) ROC curve and confusion matrix by all datasets and subgroups in the external validation (the KBASE dataset). e) all participants, f) CU participants, g) MCI participants, and h) AD participants. Abbreviation: ROC = receiver operating characteristic; AUC = area under curve, CU = cognitively unimpaired, MCI = mild cognitive impairment, AD = Alzheimer’s dementia. Supplementary table 1. Classification performance for Aβ PET positivity on ADNI and KBASE datasets. [file 13550_2021_798_MOESM1_ESM.docx]

**Supplementary figures**

**
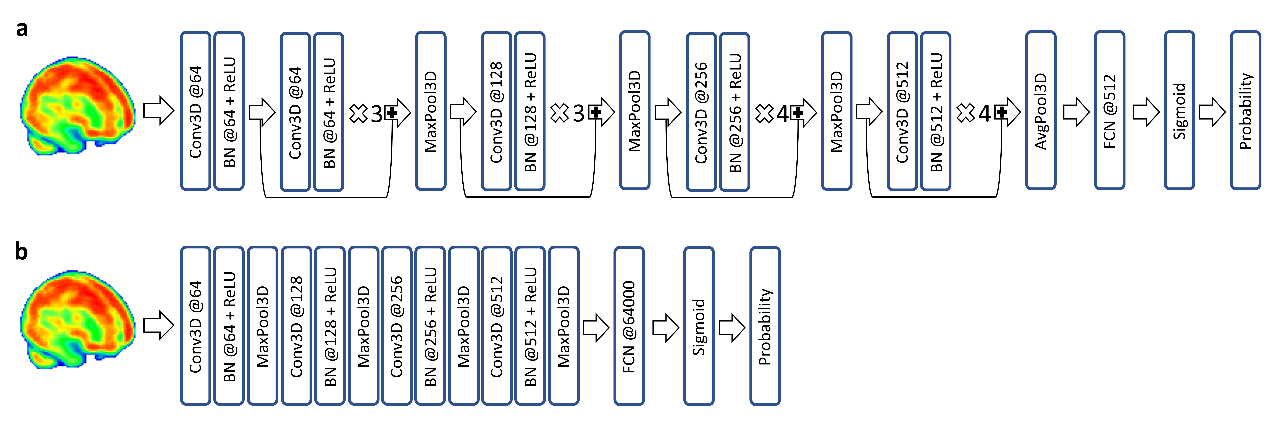
**

Supplementary Fig. 1. Convolutional neural network architecture in 3 dimensions. (3-D) a) Custom ResNet with 15 layers and 4 residual layers. b) A structure that simply changed our 2.5-D architecture to 3-D. This consist of 4 convolution layers. Abbreviation: Conv = convolution layer; BN = batch normalization; ReLU = rectified linear unit; @ = number of channels; MaxPool = Max pooling layer; AvgPool = average pooling; FCN = fully connected layer.


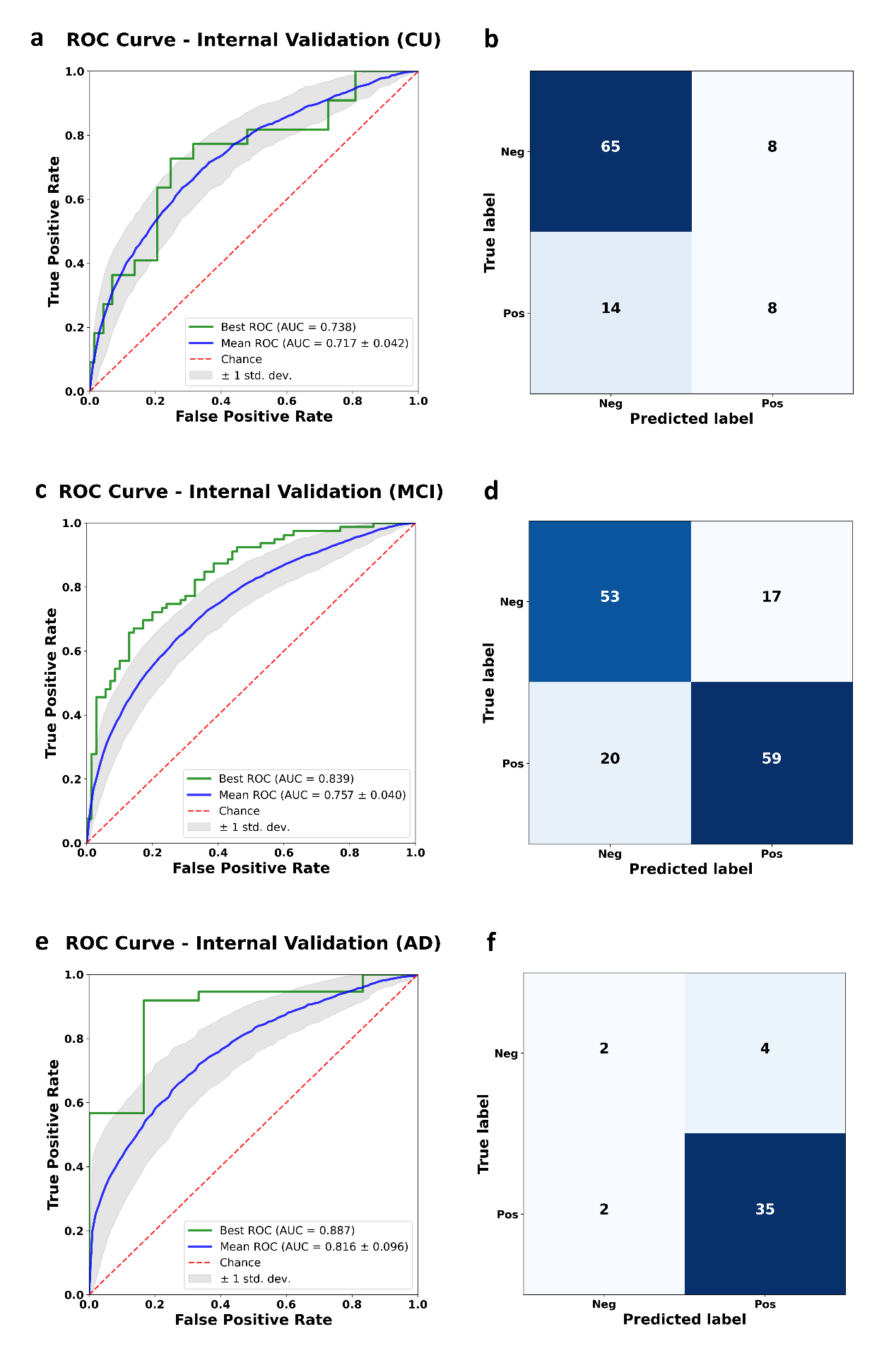


Supplementary Fig. 2. ROC curve and confusion matrix by the subgroups (CU, MCI, and AD participants) in the interval validation. (a, b) CU participants, (c, d) MCI participants and (e, f) AD participants. Abbreviation: ROC = receiver operating characteristic; AUC = area under curve, CU = cognitively unimpaired, MCI = mild cognitive impairment, AD = Alzheimer’s dementia..


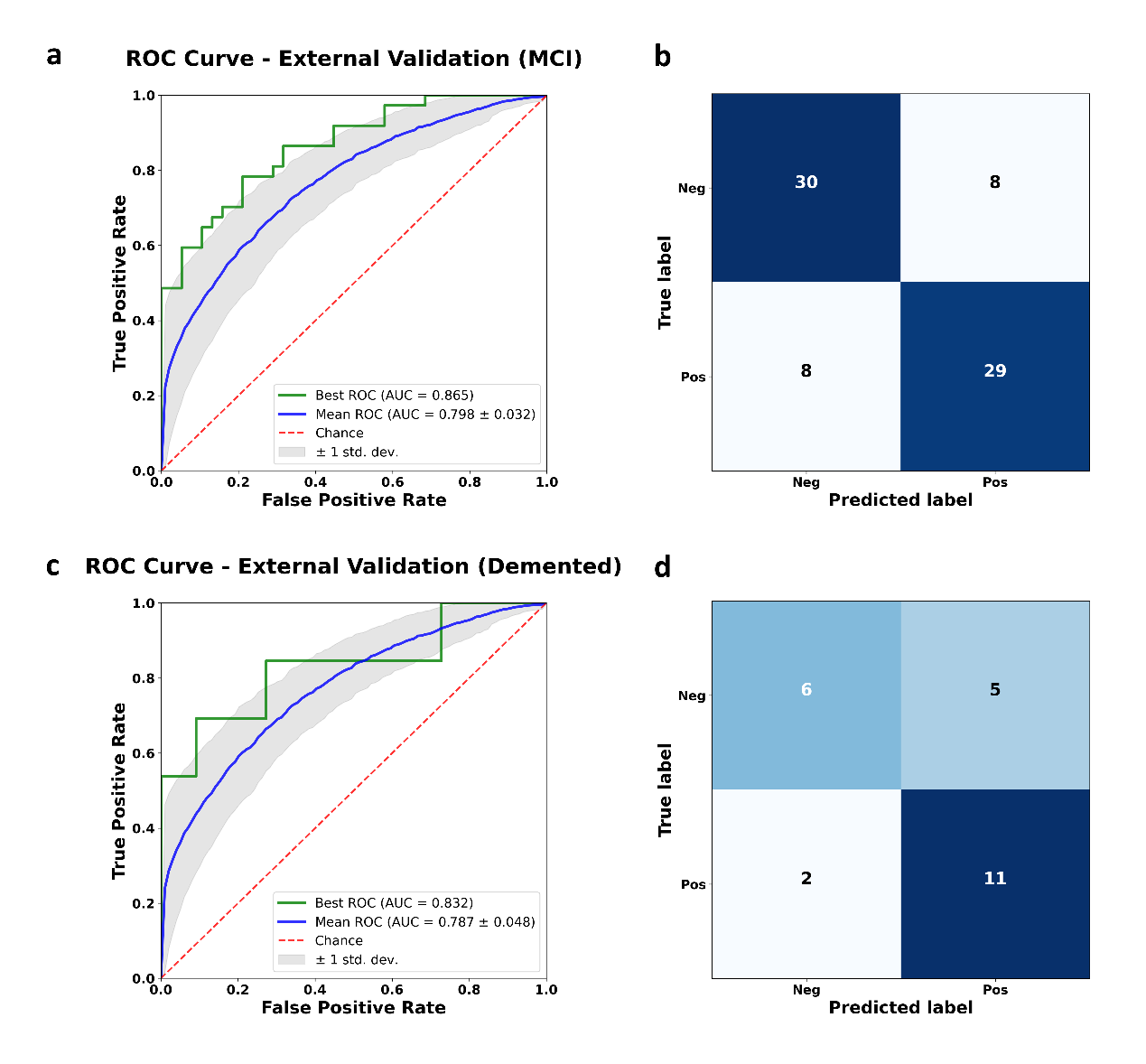


Supplementary Fig. 3. ROC curve and confusion matrix by the subgroups (MCI and demented participants) in the external validation. (a, b) MCI participants and (c, d) demented participants. Abbreviation: ROC = receiver operating characteristic; AUC = area under curve, MCI = mild cognitive impairment.


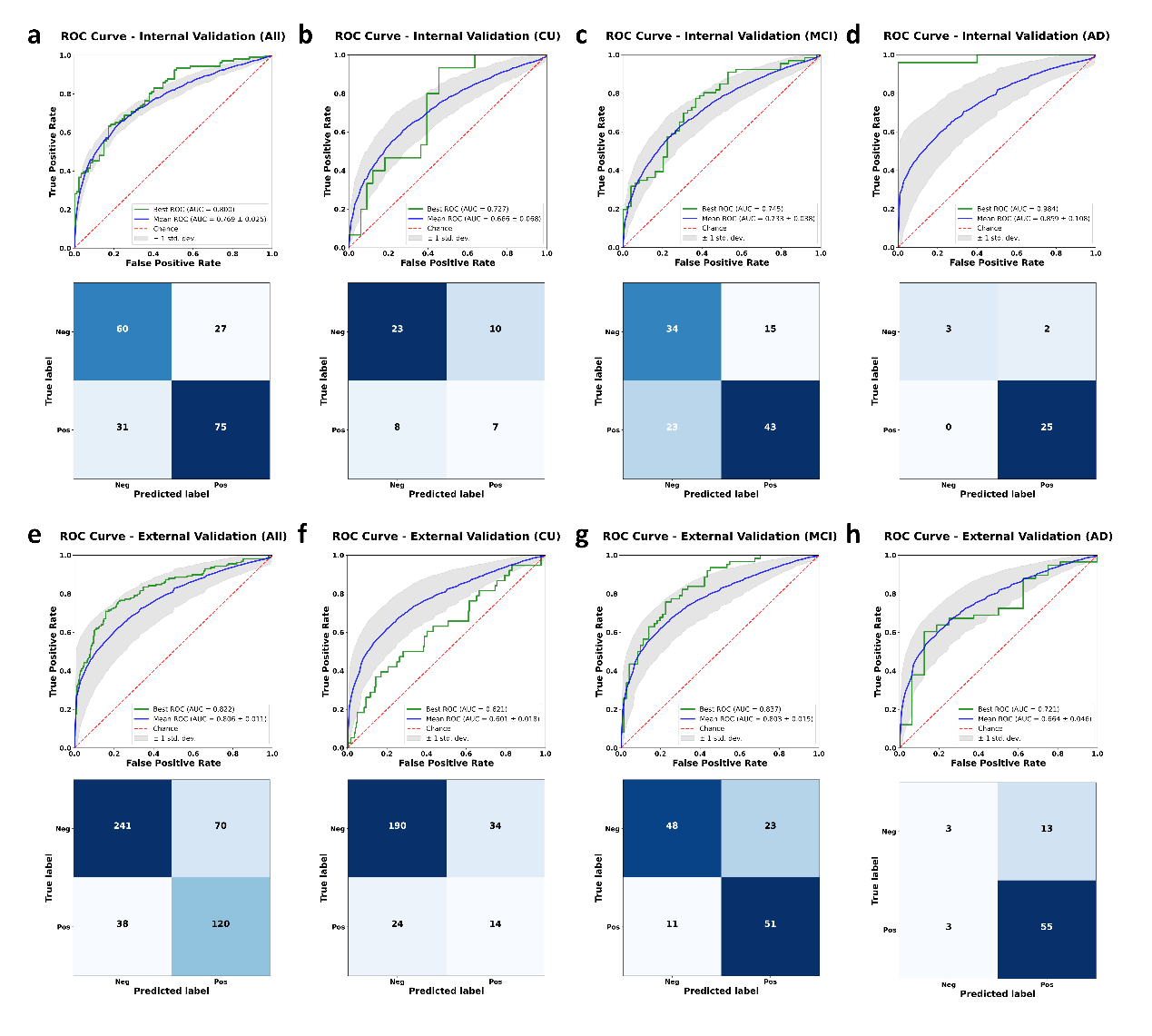


Supplementary Fig. 4. (a-d) ROC curve and confusion matrix by all datasets and subgroups in the internal validation (part of the ADNI dataset). a) all participants, b) CU participants, c) MCI participants, and d) AD participants. (e-h) ROC curve and confusion matrix by all datasets and subgroups in the external validation (the KBASE dataset). e) all participants, f) CU participants, g) MCI participants, and h) AD participants. Abbreviation: ROC = receiver operating characteristic; AUC = area under curve, CU = cognitively unimpaired, MCI = mild cognitive impairment, AD = Alzheimer’s dementia.

**Supplementary Tables**

Supplementary table 1. Classification performance for Aβ PET positivity on ADNI and KBASE datasets.

| Mean  (95% CI) | | | AUC value | | Accuracy | | Sensitivity | | Specificity | | F1-score | |
| --- | --- | --- | --- | --- | --- | --- | --- | --- | --- | --- | --- | --- |
| Internal validation (Part of the ADNI) | All | 0.769  (0.762, 0.776) | | 0.698  (0.691, 0.705) | | 0.702  (0.688, 0.715) | | 0.694  (0.675, 0.714) | | 0.718  (0.711, 0.725) | |  |
|  | CU | 0.666  (0.647, 0.685) | | 0.660  (0.642, 0.677) | | 0.507  (0.468, 0.546) | | 0.742  (0.716, 0.769) | | 0.501  (0.473, 0.530) | |  |
|  | MCI | 0.733  (0.723, 0.743) | | 0.666  (0.655, 0.677) | | 0.649  (0.632, 0.667) | | 0.690  (0.667, 0.712_ | | 0.685  (0.673, 0.697) | |  |
|  | AD | 0.859  (0.829, 0.889) | | 0.898  (0.885, 0.911) | | 0.982  (0.975, 0.989) | | 0.321  (0.246, 0.395) | | 0.943  (0.936, 0.951) | |  |
| External validation (KBASE) | All | 0.806  (0.803, 0.809) | | 0.599  (0.573, 0.624) | | 0.868  (0.850, 0.886) | | 0.462  (0.414, 0.509) | | 0.598  (0.587, 0.609) | |  |
|  | CU | 0.601  (0.596, 0.606) | | 0.536  (0.498, 0.575) | | 0.609  (0.563, 0.655) | | 0.524  (0.471, 0.576) | | 0.278  (0.272, 0.284) | |  |
|  | MCI | 0.803  (0.799, 0.807) | | 0.626  (0.610, 0.643) | | 0.929  (0.914, 0.944) | | 0.362  (0.320, 0.404) | | 0.700  (0.694, 0.707) | |  |
|  | AD | 0.664  (0.651, 0.677) | | 0.770  (0.767, 0.772) | | 0.972  (0.968, 0.977) | | 0.035  (0.022, 0.048) | | 0.869  (0.867, 0.870) | |  |

Abbreviations: Aβ = β-amyloid; CI = Confidence interval; AUC = Area under curve; CU = cognitively impaired; MCI = mild cognitive impairment; AD = Alzheimer’s dementia.
